# Identification of ultrasound imaging markers to quantify long bone regeneration in a segmental tibial defect sheep model *in vivo*

**DOI:** 10.1038/s41598-020-70426-y

**Published:** 2020-08-12

**Authors:** Songyuan Tang, Peer Shajudeen, Ennio Tasciotti, Raffaella Righetti

**Affiliations:** 1 Department of Electrical and Computer Engineering, Texas A&M University, College Station, 77843 USA; 2 Houston Methodist Hospital, Department of Orthopedics and Sports Medicine, Center for Musculoskeletal Regeneration, Houston, 77030 USA

**Keywords:** Diagnostic markers, Regenerative medicine, Biomedical engineering

## Abstract

The healing of large bone defects has been investigated for decades due to its complexity and clinical relevance. Ultrasound (US) methods have shown promise in monitoring bone healing, but no quantitative method to assess regenerated bone morphology in US images has been presented yet. In this study, we investigate new US morphometric parameters to quantify bone regeneration *in vivo*. A segmental tibial defect was surgically created and stabilized in a sheep animal model. US and computed tomography (CT) imaging data were collected two months post-surgery. New bone was assessed, reconstructed and quantified from the US and CT data using 3 morphometric parameters: the new-bone bulk (NBB), new-bone surface (NBS) and new-bone contact (NBC). The distance (mm) between surface reconstructions from repeated US was $0.49\pm 0.30$ and from US and CT was $0.89\pm 0.49$. In the mid-shaft of the defected tibia, US measurements of NBB, NBS and NBC were significantly higher than the corresponding CT measurements ($p < 0.001$). Based on our results, we conclude that US may complement CT to reconstruct and quantify bone regrowth, especially in its early stages.

## Introduction

Open long bone fractures can have serious social-economic impact. Complicated by infection, they can result in delayed union, nonunion or amputation of the extremities^1–3^. Although in the past decades marked progress in long bone fracture patient care has been made^1,4–6^, insufficient healing occurs in 5–10% of the total fracture cases^6,7^. Tibial diaphysis is a common site of fracture and segmental defect^8^ with a high risk of delayed union^9,10^. In such situations, there is an ever-increasing demand to optimize the healing outcome, following surgical treatment and through post-operative management. In cases of a severe open fracture, it is important that not only full bony bridging but also complete recovery of functional integrity occur^4^. Moreover, for weight-bearing bones like tibiae, the speed of the defect repair is critical to patients^6^, especially those under a pressing need to return to their routine duties. Thus, regenerative materials have received great interest for rapid healing of segmental defects of weight-bearing bones^11–13^. In order to evaluate the performance of new materials, provide a timely feedback on the osteo-regenerative response and clinically assess the endpoint of the healing process, medical imaging techniques are critically important.

To date, the most commonly used orthopedic imaging methods are X-ray and computed tomography (CT). Ultrasound (US) is a safe, fast and portable imaging modality. US has been employed to monitor long term fracture healing in a number of studies^14–16^, which have demonstrated that US is sensitive to changes at various stages of bone growth^14,15,17^. It has been reported in several independent limb lengthening studies that US can detect new bone formation 4-16 weeks before its appearance in radiography^18,19^. Semi-quantitative analysis reported in similar studies also indicated US to have higher sensitivity than plain radiography in monitoring early ossification events^20^. In the field of quantitative ultrasound (QUS), techniques have been developed to monitor bone formation following a fracture by measuring changes in the ultrasonic propagation velocity and attenuation^21^. Several quantitative markers were found to be closely associated with bone mineral density, microstructural and mechanical properties^22,23^.

In general, accurate and quantitative assessment of newly formed bone *in vivo* remains a challenging task. X-ray imaging has been extensively used for evaluation of clinical bone healing^11,12,24,25^, but it primarily provides a qualitative assessment. CT provides exquisite 3-D visualization of the defected bony site and allows various types of quantification such as bone volume^12,26,27^ and density^25^. However, it is expensive and requires relatively high ionizing radiation doses, which make it unsuitable for frequent monitoring of bone growth^28^. In the realm of histomorphometry, parameters such as bone surface (BS), implant surface and mineralizing surface (MS/BS) have been used as markers in different *ex vivo* and *in vitro* bone regeneration/metabolism studies^11,29,30^. Although histomorphometry provides a broad choice of parameters to effectively characterize bone activity at the cellular level, it works only on tissue samples extracted at specific locations. Hence, it is not able to fully profile the massive bone formation in 3-D space. Moreover, in a clinical setting, assessment and quantification of new bone using histomorphometry require bone biopsy for the procurement of testing samples. In comparison, US is cost-effective, does not require anesthesia and allows non-invasive imaging of a large portion of bone surface without using radiations. These characteristics make US an ideal imaging modality in pediatric applications and for the assessment of a wide variety of regenerative materials^28,31^. In this context, existing QUS methods have a number of limitations such as substantial variations in the experimental setup and protocols, which can make their clinical translation challenging^21,23,32^. By contrast, evaluation of bone morphology from US scans is generally robust to variations in the acquisition protocol, and US scans for bone 3-D imaging are fast and widely deployed. Thus, US-based bone morphometry would be easier to standardize and would more objectively reflect regeneration outcomes. While the use of US to assess regenerated new bone morphology is currently limited to semi-quantitative analysis^20^, previously-developed US-based bone surface detection techniques^33,34^ may still be effective in detecting newly formed bone in 3-D. Such techniques could be coupled with markers of tissue morphometry to accurately quantify bone formation from the US data.

In this paper, we present the first study in an *in vivo* segmental tibial defect sheep model aiming at the identification of US imaging markers that can be used to quantify new bone growth. This defect was surgically created and stabilized using a poly-ester urea (PEU) shell^35^. Three morphometric parameters indicative of the bulk, surface and contact of the new bone deposited on the shell were defined and computed from US images. Independent US data sets and benchmark CT were used to demonstrate method repeatability and compare performance across the two modalities.

## Results

**Figure 1:**
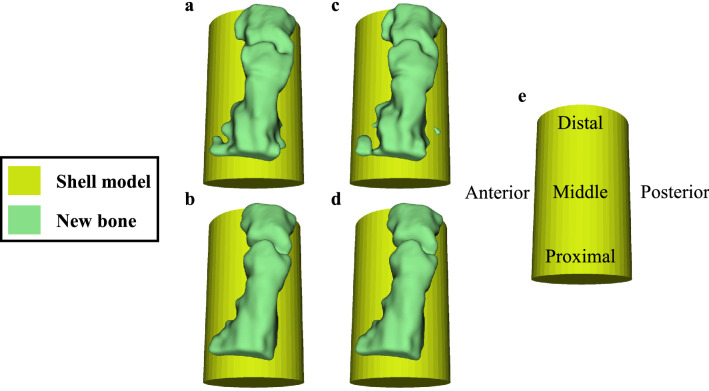
Illustration of the new bone bulk volume reconstruction from US (row 1) and benchmark CT (row 2) from 2 acquisitions (**a**) and (**c**). (**e**) Shows the relative anatomical location of the shell model in the sheep leg. Smoothing was applied equally to new bone reconstructions in this figure.

**Figure 2:**
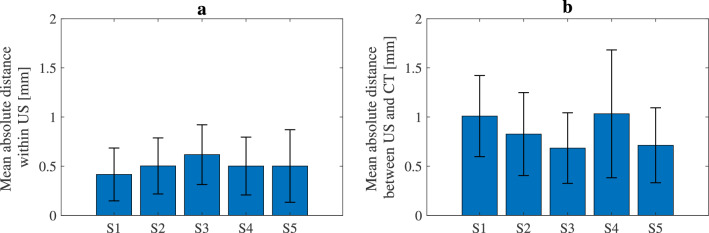
Bar plots of the mean absolute distance in the new bone surface from 2 US scans (**a**) and from US and CT scans (**b**) respectively.

**Figure 3:**
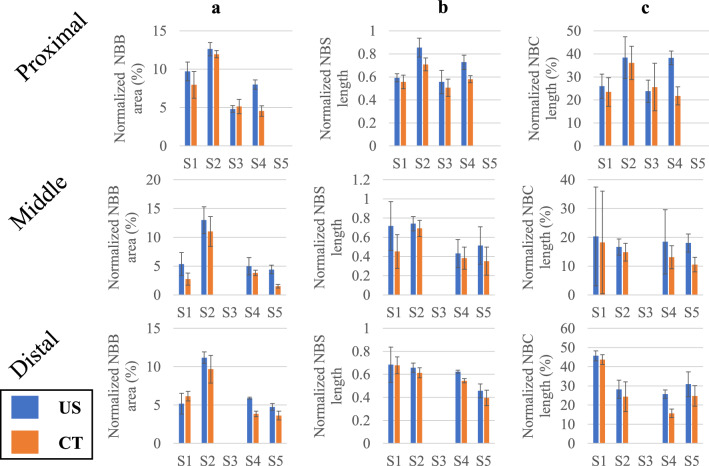
Bar plots of the normalized NBB (**a**), NBS (**b**) and NBC (**c**) from US and CT measurements from the proximal (row 1), middle (row 2) and distal (row 3) portion of the sheep leg.

**Figure 4:**
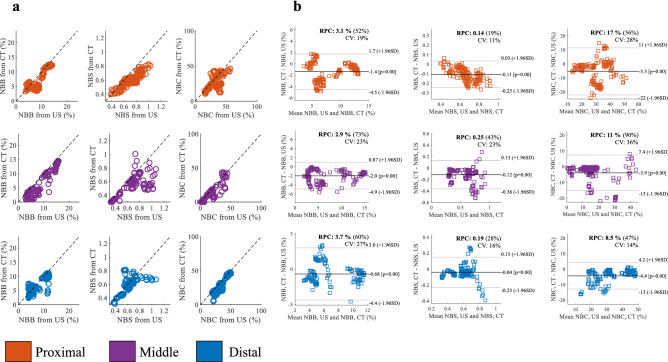
Comparison of morphometric parameters derived from US and from CT in the proximal, middle and distal regions: (**a**) Scatter plot and (**b**) Bland-Altman plot.

In this section, we present the results of our quantitative analysis, namely the new-bone bulk (NBB), new-bone surface (NBS) and new-bone contact (NBC) morphometric parameters computed using US data obtained from 5 sheep (S1-S5). From each sheep, the parameters were obtained from each axial plane in the volume of interest (VOI) and are presented here as mean ± standard deviation (SD). For the purpose of illustration, we first show one set of spatially aligned US/CT data compounded in the same VOI for a selected sheep (S2, see also supplementary video). For the same sheep, we also show the 3-D reconstruction of its NBB geometry from 2 US scans across approximately the same areas together with their CT benchmarks (Fig. 1). We note the similarity between the detected bone surfaces from the 2 US scans demonstrating repeatability in the US scans as well as from the US and CT scans (Fig. 2). We then compare the three morphometric parameters obtained from all US scans (across all sheep and across the entire VOI, Table 1) and from US and CT scans (for each individual sheep, Fig. 3, and across all sheep, Table 2 and Fig. 4, both shown in the proximal, middle and distal portions respectively). Morphometric parameters across all sheep were analyzed using the method of Bland and Altman[^36,37^].

**Table 1:** Mean, standard deviation (SD) and 95$\%$ limits of agreement of the difference in the normalized NBB, NBS and NBC between 2 US measurements.

Normalized NBB area ($\%$)			Normalized NBS length			Normalized NBC length ($\%$)		
Mean	SD	$95\%$ limits of agreement	Mean	SD	$95\%$ limits of agreement	Mean	SD	$95\%$ limits of agreement
0.18	0.93	$-1.65$, 2.00	$-0.01$	0.08	$-0.17$, 0.15	0.22	4.24	$-8.09$, 8.53

**Table 2:** Mean, standard deviation (SD) and 95$\%$ limits of agreement of the difference in the normalized NBB, NBS and NBC between US and CT measurements from the proximal (P), middle (M) and distal (D) portion of the sheep leg. Positive mean values indicate higher CT measurement than US.

	Normalized NBB area (%)			Normalized NBS length			Normalized NBC length (%)		
	Mean	SD	$95\%$ limits of agreement	Mean	SD	$95\%$ limits of agreement	Mean	SD	$95\%$ limits of agreement
P	$$-1.37***$$	1.59	$$-4.49,1.74$$	$$-0.11***$$	0.07	$$-0.25,0.03$$	$$-5.33***$$	8.55	$$-22.10,11.43$$
M	$$-2.02***$$	1.47	$$-4.91,0.87$$	$$-0.12***$$	0.13	$$-0.36,0.13$$	$$-3.89***$$	5.78	$$-15.21,7.43$$
D	$$-0.68***$$	1.88	$$-4.37,3.00$$	$$-0.04***$$	0.10	$$-0.23,0.15$$	$$-4.35***$$	4.35	$$-12.89,4.18$$

$$^{***}{p}<0.001$$, two tailed Student’s t-test.

### New bone visualization and reconstruction

The volumetric reconstructions of the new bone from the two US scans are in good qualitative agreement (Fig. 1a,c). When the compounded US/CT data as well as their respective reconstructions are visually examined, the overall similarity in the new bone between the US and CT images is noticeable (supplementary video, Fig. 1a–d). The detected new bone surfaces from the 2 US scans exhibit an average mean absolute distance ranging from 0.42 mm to 0.62 mm (Fig. 2a sheep S1, S3), while that from US and CT scans show an average mean absolute distance ranging from 0.68 mm to 1.03 mm (Fig. 2b sheep S3, S4). Across all sheep, the mean of the mean absolute distance between repeated US and between US and CT is 0.49 mm (SD: 0.30 mm) and 0.89 mm (SD: 0.49 mm), respectively.

### Morphometric parameters

When the 3 morphometric parameters are compared across US scans, the mean difference in the normalized NBB area and its 95% limits of agreement are 0.18% ($-1.65\%$, 2.00%), while those in the normalized NBS and NBC length are $-0.01 (-0.17, 0.15)$ and 0.22% $(-8.09\%, 8.53\%)$, respectively (Table 1).

To compare the 3 morphometric parameters obtained from US and CT scans, we report the individual results obtained in the proximal, middle and distal regions. Figure 3 shows this comparison for each individual sheep, whereas Fig. 4a compares each parameter derived from US and CT lumped for all sheep. From both plots it can be noticed that values of parameters computed from US data are in general higher than the corresponding ones obtained from CT data. Table 2 summarizes the statistical results obtained across all sheep. The proximal portion of the VOI shows a mean difference (CT-US) and 95% limits of agreement in the normalized NBB area to be $-1.37\% (-4.49\%, 1.74\%)$, normalized NBS length to be $-0.11 (-0.25, 0.03)$ and normalized NBC length to be $-5.33\% (-22.10\%, 11.43\%)$. The middle portion of the VOI shows a mean difference and 95% limits of agreement in the normalized NBB area to be $-2.02\% (-4.91\%, 0.87\%)$, normalized NBS length to be $-0.12 (-0.36, 0.13)$ and normalized NBC length to be $-3.89\% (-15.21\%, 7.43\%)$. As for the distal portion of the VOI, the mean difference and 95% limits of agreement in the normalized NBB area are $-0.68\% (-4.37\%, 3.00\%)$, normalized NBS length are $-0.04 (-0.23, 0.15)$ and normalized NBC length are $-4.35\% (-12.89\%, 4.18\%)$. Two-tailed Student’s t-test indicates all parameters to be significantly larger in US than in CT, for all regions of the VOI investigated ($p < 0.001$). Figure 4b shows the corresponding Bland-Altman plots.

## Discussion

In this paper, we present three new US imaging markers that can be used to quantify new bone formation. As a testing platform, these markers are evaluated in a segmental tibial defect sheep model. Segmental tibial defects are of strong clinical relevance as they often arise in a variety of clinical situations such as large tumor removal, motor vehicle accidents as well as blast injuries^38^, and their related post-traumatic management is difficult to optimize^39^. Recent technology concentrates on the development of new bone graft substitutes to explore better solutions to the crux of large bone defect nonunion^11,40,41^. In related preclinical studies, the sheep model is commonly used owing to the similarities between humans and sheep in body weight and tibia dimension^38,42^. Thus, implants designed for human applications are commonly tested in pilot sheep studies^43^.

Diagnostic imaging is required to monitor the course of fracture healing^6^. As of today, the basic modality to evaluate fracture consolidation remains projection radiography^6,11,12,24,25,38,44,45^. In cases where conventional radiograms fail to reveal the healing status of the bony defect, CT can be performed to accurately 3-D reconstruct the fracture zone^6^. US has been shown to complement radiography in imaging newly formed bone as well as several types of bone fractures and defects^14,15,17,31,46,47^. Availability of automatic bone surface detection techniques^33,34,48^ can further extend the successful use of US in orthopedic applications. While a large body of past work concentrated on qualitative or semi-quantitative US evaluation^14–16,20^, there remains a lack of objective quantification tools to assess healing outcomes in early fracture stages. Current QUS techniques lack standardization among manufacturers, leading to inconsistent imaging markers^21–23,32^. To the best of our knowledge, this paper presents the first US study focusing on imaging and reconstruction of morphometric parameters in long bone regeneration *in vivo*. With respect to earlier US bone evaluation methods, this method has the following benefits: 1) it allows assessment of the new bone formation in 3-D; 2) the scan is rapid, easy to perform and can be performed using commonly available US scanners and 3) it is robust to variations in acquisition parameters. In the future, 3-D US imaging techniques may be used alone or in conjunction with CT to non-invasively quantify bone regrowth with high sensitivity, high reliability and at minimized patient radiation dose.

From a quantification point of view, the new bone mass and surface area are parameters of particular interest^11,12,25–27,29,30,49,50^. In the presence of an implant, both the outer surface (i.e., NBS) and contact surface (i.e., NBC) have been used to characterize new bone formation^11,30^. In this study, NBB was used to approximate the amount of new bone mass deposited on the shell surface, which is typically measured from CT. The new bone mass is presumably the most direct indicator of osteoregeneration - the larger this value is, the more effective the material is in inducing/forming new bone. Therefore, it is a common type of quantification performed in assessing tissue-engineered grafts^12,26,27^. Due to limited US penetration in bones, this surrogate measure of new bone mass was approximated in the bulk sense (hence the name “new-bone bulk”). This bulk measure ignores non-bone structures such as neo-vessels or soft callus that may reside underneath the bone surface, especially at the early stages of bone regeneration. Similar accommodations are used when quantifying the callus area or bone volume using radiography^44,50^. NBS and NBC, on the other hand, were inspired from histomorphometry. In the past, neither of these parameters could be measured non-invasively and *in vivo*, making the quantification possible either at one single time point or through repeated invasive procedures. Thanks to the physical principles underlying US imaging, the NBS parameter is relatively easy to extract from B-mode images. On the basis of bone histomorphometry^51^, NBS was introduced here to measure the spatial extent of the healing bone. In some sense, this parameter functions similarly to MS/BS from bone histomorphometry in the application of regenerative materials, despite the distinct scales they manifest at. MS/BS quantifies the new bone through labeling agents that act on the site of forming bone^52^, whereas, in US imaging, large mismatch in acoustic impedance creates a contrast mechanism to localize surfaces^53^. However, due to the large attenuation inside bones, NBS was presented in this study as a marker of new bone surface detected on the outermost envelope of the healing bone. Also, the correlation between acoustic impedance and degree of mineralization needs to be studied more systematically to aid in the understanding of the nature of surfaces that can be detected using US. As far as NBC is concerned, it was derived from NBS by imposing additional assumptions of complete ossification radially underneath the envelope of new-bone surface and in the vicinity of the shell surface. Under this assumption, NBC provides an interpretation of osteointegration. For example, in the application of oral implants where osteointegration is defined as a direct structural and functional connection between ordered, living bone and the surface of a load-carrying implant^54^, parameters such as the bone-to-implant contact (BIC) have been identified as key factors to a long-term success of the implant^30^. BIC measures the linear surface of the implant directly contacted by the bone matrix, and is often expressed as a percentage of the total implant surface^30^. In the specific context of dental histomorphometry, such a contact is examined at the microscopic level^55^ with the requirement of interposed soft tissue to be absent at the interface^56^. However, an overall peri-implant stability, which may allow more feasible means of assessment, is gaining momentum in a clinical setting^56^. Consequently, the NBC parameter obtained from US could be one practical imaging marker to assess scaffold osteointegration and defect stability. Of note, due to the assumptions used in our derivation, the NBC and NBB should be interpreted as the upper limit of the actual BIC and new bone mass. Low NBC and NBB values will invariably imply low BIC and new bone mass, hence “poor” new bone formation. In this paper, the nomenclature for the morphometric parameters, i.e., NBB, NBS and NBC, is left dimension-indefinite. As we present these parameters in 2-D axial planes for the convenience of statistical comparison, they are referred to as “NBB area”, “NBS length” and “NBC length”. In principle, these parameters can also be measured in sagittal, coronal planes or in 3-D, but the interpretation of their physical meaning may be different. In the 3-D case, in particular, they should be referred to as “NBB volume”, “NBS area” and “NBC area”. NBB, NBS and NBC are generic indicators of new bone formation and can be used to assess the performance of different implants in osteogenesis and in a clinical setting to monitor fracture healing.

For the comparison between US and CT results, the threshold of CT data was determined from the region where native bone is not present. This enables the use of standard thresholding methods in orthopedic applications^57^. Overall, remarkable visual agreement between 3-D geometries reconstructed from US and CT was observed (see Fig. 1).

When new bone surfaces obtained from 2 repeated US scans were compared, the mean absolute distance was observed to be no more than 1 mm (in an average sense). This dissimilarity primarily arises from small misalignment in the US data, as well as variation in the response of the bone surface detector at different insonification angles. However, similar error values were found in previous work on segmenting the bone surface from US images of human patients, and was confirmed to fall within the tolerance range^58^. This is also visually corroborated by the conspicuous similarity between a and c in Fig. 1. It is noteworthy that US imaging artifacts such as refraction, deflection and speed calibration errors tend to result in inaccurate bone surface profiles^59^, which was found to be most evident in the posterolateral and anterolateral aspects of the tibia/shell. This can be noted in a and c of Fig. 1 where a flat layer lateral to the bridged bony collars toward the anterior or posterior aspect is interpreted as the new bone. Nonetheless, this inaccuracy is relatively small.

When the 3 morphometric parameters are compared between the US and CT measurements, it can be seen from Fig. 3 that differences generally exist in all parameters despite the overall congruence shown in Fig. 2. This result is expected as US and CT generate images of the new bone based on distinct physical principles and hence may exhibit different sensitivity to specific contents of new bones. According to our results, the middle portion of the VOI demonstrates the most pronounced difference between US and CT in terms of NBB and NBS. Table 2 confirms a significantly higher average value in all three US-based morphometric parameters. This observation appears to confirm earlier findings^15,18,19^, where US was demonstrated to visualize new bone formation before its appearance in radiography.

Quantification in this paper is limited to the field of view (FOV) of each sheep’s US scan. In the future, multiple views of US scans may be fused to obtain a single volumetric representation^60–62^, and position sensors may be used in the absence of CT data for spatial calibration. In the situation where a tracker is not available, internal tracking with image features can be used. In particular, speckle decorrelation has been reported as a reliable feature to infer separation between US frames obtained from linear array transducers^63–65^. If 2D arrays were to be used, then the relative location of the frames within each single volume would be automatically known and no or minimal motion of the probe would be required to instantaneously collect volumetric data. Therefore, performance of tracking in large FOVs would be improved. In addition to morphometric parameters, bone vasculature is also a key diagnostic and prognostic factor as vessels provide effective means to supply nutrition, exchange gas as well as egress break-down products^66^. Currently, bone vascularization is identified microscopically by labeling vessel walls or highlighting the lumen using contrast agents^67^. Within the US imaging field, power Doppler and dynamic contrast-enhanced (DCE) US have been used primarily in the context of tumor vascularity^68^. Laser Doppler flowmeters have been applied to measure the blood flow across a fracture gap^69^. For large animals, the role of vessels in bone remodeling may be different from that in small animals^67^, and the corresponding imaging methods may require necessary adjustment. Of note is the typical high imaging frequency (20MHz-100MHz) required to resolve microvasculature. Hence, its adaptation to large animal models will need to consider the resolution/penetration trade-off. In this study, US scans were performed in the large animal model 2 months post-op. As this time window is not ideal for observing neo-vessel formation^66^, early indicators of bone healing propensity such as angiogenesis will need to be investigated in the future with a dedicated study in a smaller animal model. This cross section study may also be extended to a longer time range. In such a context, 3-D morphometric parameters would be of great interest to fully investigate the mechanism of large bone defect healing as well as to evaluate the performance of specific regenerative materials in stimulating the healing process.

## Conclusions

In this paper, three US imaging markers that can be used to quantify bone regeneration morphometry were extracted from a segmental tibial defect sheep model. The results show agreeable repeatability within US and consistency between US and CT in visualization and reconstruction of new bone geometry in 3-D. Morphometric parameters from US scans further show significantly higher average values with respect to benchmark CT. These results provide the first quantitative comparison between US and CT in evaluating new bone formation and demonstrate that US may complement CT to reconstruct and quantify bone regrowth, especially in its early stages. The presented framework of quantification can have a significant impact on the field of regenerative materials and, in general, diagnostic imaging of bone fracture healing.

## Materials and Methods

### Animal treatment and data acquisition

This study was approved by the Houston Methodist Research Institute Institutional Animal Care and Use Committee (IACUC) (study#: AUP-1215-0072, Award W911NF-11-1-0266). All experiments were performed in accordance with relevant guidelines and regulations. Five female sheep aged approximately 24 months were used. For each sheep, osteotomy segmental defects (3 cm) were made in the tibia by creating an incision on the anterior side of the hind leg, disconnecting the periosteum from the diaphyseal tibial portion and removing a bone segment of the prescribed size. A rigid shell (total length $\approx$ 7 cm) was then used to connect the bone fragments and maintain the interfragmentary gap size. After the surgery, immobilization of the leg motion was accomplished by means of a plaster splint.

Ultrasound evaluation was performed around 2 months post-operatively. Prior to the scan, the sheep was lying in a recovery position under anesthesia. In order to ensure adequate ultrasound wave transmission, the leg surface was shaved. All experiments were performed using a 38-mm linear array transducer (Sonix RP, Ultrasonix, Richmond, BC, Canada) with 128 elements, 5-14 MHz bandwidth, 50% fractional bandwidth at -6dB and 1 mm beamwidth at the focus^32,70,71,72,73^. The center frequency was set at 10 MHz and depth was 3.5 cm or 4 cm. The region of interest (ROI) was the lateral side of the leg. As the newly formed bone surface may have a wider depth range, 3 focal zones were used to optimize imaging quality at the bone surface. Cine-loop data were then recorded while the transducer probe was moved along a linear path on the skin surface. The CT imaging protocol used in this study has been reported in earlier papers^74,75,76^.

### Pre-processing and object detection

For computation of the morphometric parameters to quantify the bulk, surface and contact of the new bone, knowledge of the new bone representation in US and CT together with knowledge of the shell is required. For detecting new bone in the US data, the B-mode images were pre-processed with noise suppression and bone surface enhancement techniques as in our previous study^34^. A method based on the 3-D phase symmetry (PS)^48^ was then applied to identify the bone surface candidates. In particular, the 3-D Log-Gabor filter was constructed to detect the phase symmetry. This filter bank contained a single scale and a single orientation due to the general level surface posited by long bone surfaces. The PS response was fed into a local phase symmetry model^48^ to augment bony surface features. The output response image was further binarized (voxel value 1 for the foreground and 0 for the background). Isolated structures falling on the image borders were removed as well as tissue structures in the skin layer and surrounding the lumen of blood vessels. Portions of the segment delineating the shell surface was also removed to avoid ambiguity in the shell surface definition. Finally, small remnants were cleared using an area opening filter. The geometry of the shell was determined from the CT data by identifying landmark points in the outer rim of the radio-lucent region and constructing a cylindrical shape model. Whereas new bone from the CT data was obtained by setting a threshold^27^ for each sheep. This threshold was determined from the defected zone where native bone was absent. More specifically, the CT intensity values across this defected zone and within the region of soft tissue were extracted, and the Otsu threshold was calculated from its distribution. Prior to thresholding, the CT image was smoothed using a Gaussian filter. Similar to US, small remnants in the CT segmentation were cleared using an area opening filter.

The VOI for the detected objects was determined as the lateral aspect of the sheep leg that did not exceed the lengthwise range of the shell. In this VOI, all detected objects were manually aligned, reformatted and binarized in the same 3-D space and exported as a series of axial slices. Pre-processing and object detection were performed using 3D Slicer 4.10.1 (Brigham and Women’s Hospital, Boston, MA).

**Figure 5:**
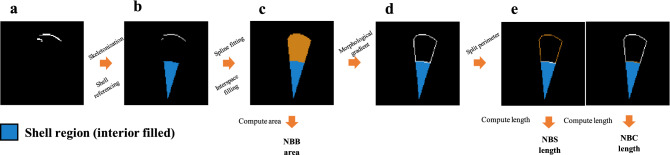
Schematic of US image post-processing and quantification. Image contents highlighted in orange denote the new-bone bulk geometry (**c**), new-bone surface perimeter (**e**, left column) and new-bone contact perimeter (**e**, right column) used to derive the corresponding morphometric parameters.

### Post-processing and quantification

The procedure of post-processing and quantification is illustrated in Fig. 5. In each slice of the bone surface segmented from US, foreground areas were thinned and spurious branches were pruned^34^. In each column of the image, only the pixel with the lowest depth was kept to avoid ambiguity arising in partial occlusion (Fig. 5b). Next, a smoothing spline (smoothing parameter=0.5) was used to fit this profile. Interspace between this fitted curve and its radial projection onto the shell surface was filled, and its area was defined as the NBB area (Fig. 5c). The perimeter of this filled region (Fig. 5d) was split into two parts, one contained in the domain of the shell (Fig. 5e, right column) and the other complementary to it (Fig. 5e, left column). The length of the former part of the curve was defined as NBC length and the latter as NBS length. Shell surface between disjoint bone surfaces was also considered when calculating NBS. CT data were thresholded followed by extraction of the bulk region and perimeter. Derivation of the three parameters refers to that detailed for US quantification. Computation of the mean absolute distance refers to our earlier study^34^.

Following the aforementioned processing, the NBB area, NBS length and NBC length were computed from each axial slice in the VOI from 2 repeated US scans. This also provided 2 identical sets of CT benchmarks. The horizontal range of the new bone was determined as the common bone in the 2 repeated US data. Bone outside this range was not considered for the quantification. The VOI was partitioned lengthwise into 3 portions (relative to the shell’s rostral end): proximal (5%–35% of the shell length), middle (35%–65% of the shell length) and distal (65%–95%). Axial slices out of this range were not used, and slices where new bone was not detected were also not used. The NBB area was normalized relative to the area of the FOV and NBS and NBC length were normalized relative to the half circumference of each shell model’s endcap. Finally, morphometric parameters extracted from the 2 repeated US and their benchmark CT were further averaged for the comparison between US and CT. All post processing and quantification were performed in Matlab (MathWorks Inc., Natick, MA, USA) installed on the supercomputers located at the Texas A&M High Performance Research Computing (HPRC) facility.

### Statistical analysis

Two tailed Student’s t-test was used to indicate statistical significance in the differences of the morphometric parameters between US and CT ($\alpha$=5%).

## Supplementary Information

.

Supplementary Video 1

Supplementary Video 2

Supplementary Video 3

## Data Availability

The datasets generated during and/or analyzed during the current study are available from the corresponding author on reasonable request.
